# Significant efficacy and well safety of apatinib in an advanced liver cancer patient: a case report and literature review

**DOI:** 10.18632/oncotarget.14724

**Published:** 2017-01-18

**Authors:** Peisi Kou, Yan Zhang, Wenbo Shao, Hui Zhu, Jingze Zhang, Haiyong Wang, Li Kong, Jinming Yu

**Affiliations:** ^1^ Department of Radiation Oncology, The First Affiliated Hospital of Zhengzhou University, Zhengzhou, China; ^2^ Department of Radiation Oncology, Shandong Cancer Hospital Affiliated to Shandong University, Jinan, China; ^3^ School of Medicine and Life Sciences, University of Jinan - Shandong Academy of Medical Sciences, Jinan, China; ^4^ Department of Intervention, Shandong Cancer Hospital Affiliated to Shandong University, Jinan, China

**Keywords:** hepatocellular carcinoma, apatinib, targeted therapy

## Abstract

Apatinib is a novel and highly selective tyrosine kinase inhibitor of vascular endothelial growth factor receptor-2. Previous studies have suggested that apatinib is safe and effective in some solid tumors. We report one case with advanced hepatocellular carcinoma (HCC), who received apatinib combined with transhepatic arterial chemotherapy and embolization (TACE), and chemotherapy respectively. TACE was administered three times once a month, using lipiodol 10ml, oxaliplatin 150mg, and tegafur 1g. The dose of apatinib was 500 mg/d from day 4 to 24. After TACE, the patient received chemotherapy of regimen FOLFOX4, oxaliplatin intravenously at 85 mg/m^2^ on day 1, calcium levofolinate 200 mg/m^2^ on day 1 and 2, 5-fluorouracil 400 mg/m^2^ intravenously and 5-fluorouracil 600 mg/m^2^ intravenously pumped for 22h on day 1 and 2, cycled every two weeks for seven cycles. He took concurrently apatinib with a dose of 500mg daily from 1 to 10 days per cycle. He was confirmed as partial response (PR) by the Response Evaluation Criteria in Solid Tumors (RECIST). The level of serum alpha-fetoprotein (AFP) decreased from 60500 ng/ml to 12.7 ng/ml, and the progression free survival (PFS) time was more than eight months. It indicated that apatinib may be a superior choice for HCC patients.

## INTRODUCTION

Liver cancer ranks the fourth in the morbidity rate of cancers in China, and it is the third leading cause of cancer death among both men and women [1]. Therapeutic methods of liver cancer contain surgery, interventional therapy, radiofrequency ablation, microwave ablation, chemotherapy, radiation therapy, targeted therapy and liver transplantation. Nowadays, with the development of precision medicine, targeted therapy gained more and more attention.

Apatinib is the first generation of oral antiangiogenesis drug. As the tyrosine kinase inhibitor of vascular endothelial growth factor receptor-2, apatinib could prevent the growth of tumor. Some clinical trials have proved the effect of apatinib on advanced gastric cancer [2, 3] and hepatocellular carcinoma (HCC) [4]. Here we report one case using apatinib combined with transhepatic arterial chemotherapy and embolization (TACE) and chemotherapy on treatment of liver cancer in our hospital.

## CASE REPORT

**Figure 1:**
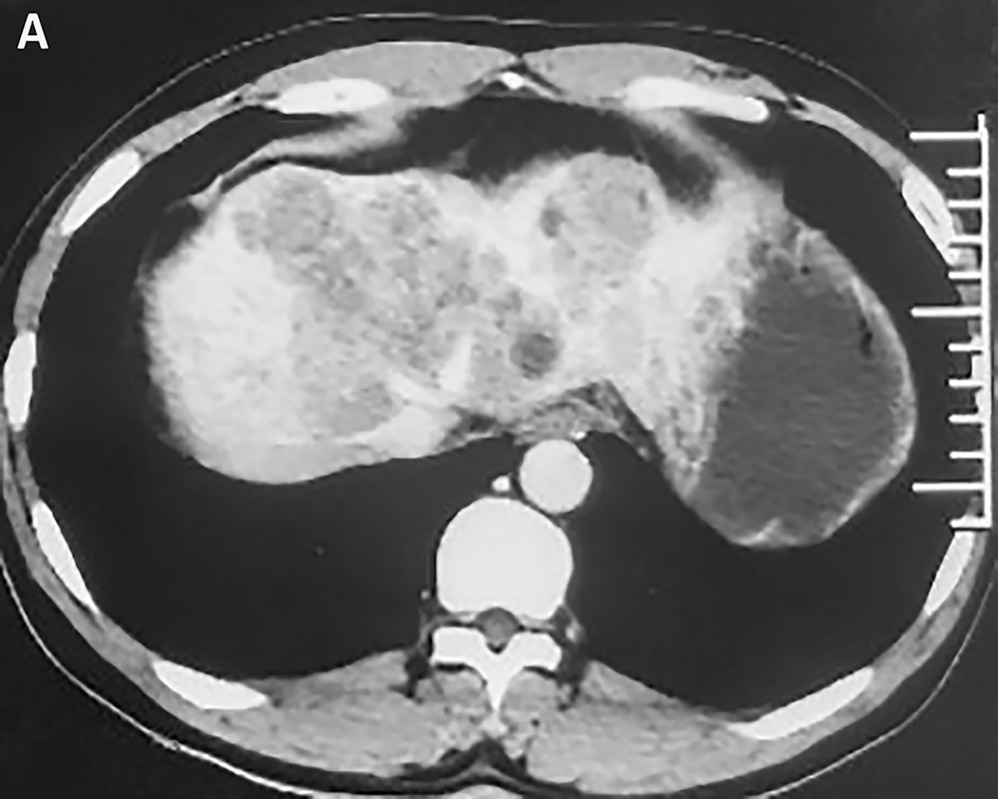

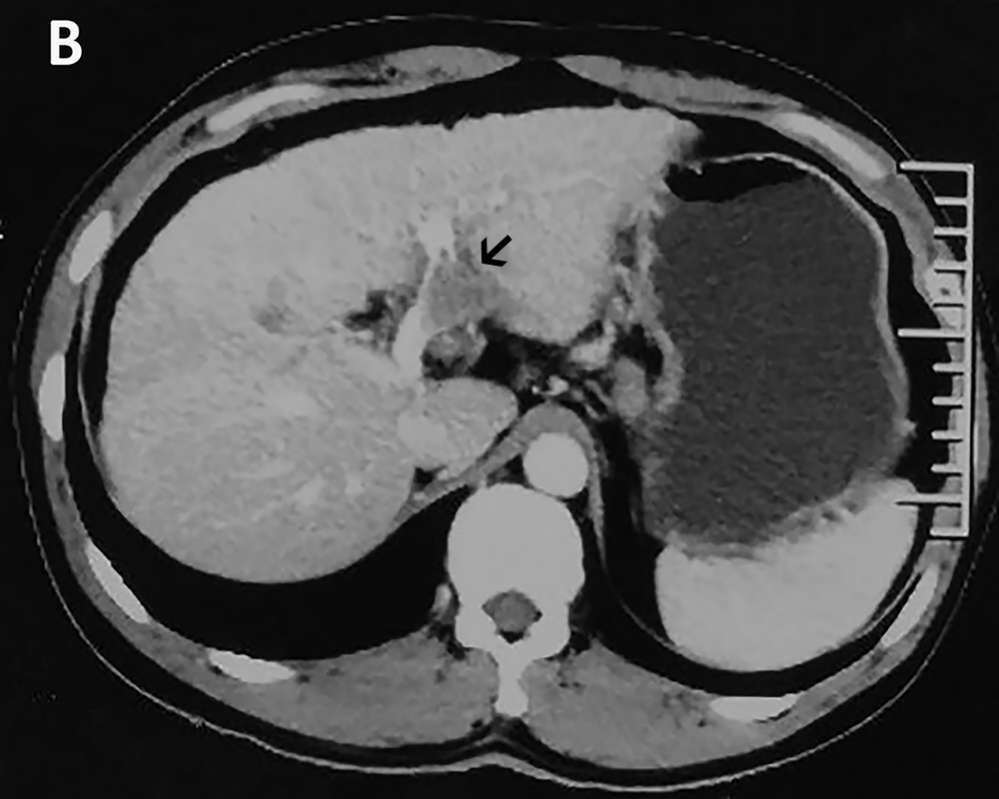
Abdomen CT images show that one of the lesions is located in the top of liver **A**. In the venous phase, the mass is low density and irregular. **B**. The arrow represents tumor thrombus in the left branch of portal vein.

**Figure 2:**
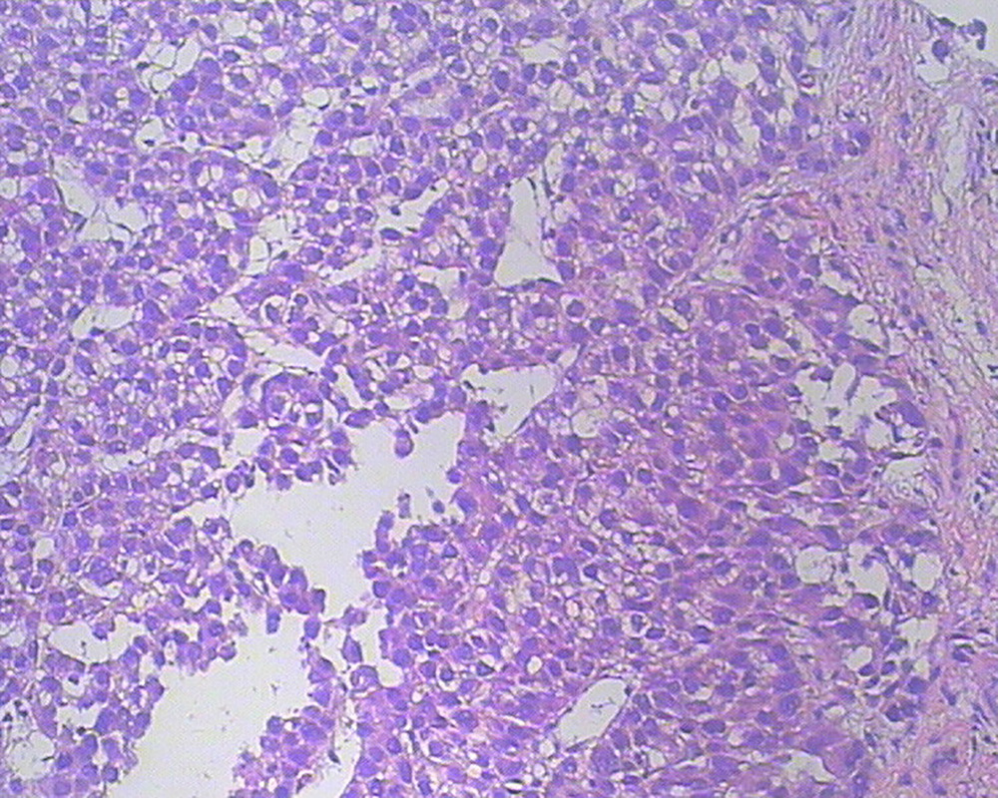
Hematoxylin and eosin staining of a tumor section (×200) The pathological diagnosis is HCC.

In November 2015, a 45-year-old man was referred to our hospital with complains of diarrhea and fever for one month. He had a history of chronic hepatitis B for more than ten years and drank for twenty years, but no family history of hepatitis and cancers. Physical examination showed no positive sign, and Eastern Cooperative Oncology Group (ECOG) performance status was 1. The concentration of serum alpha-fetoprotein (AFP) exceeded 60500 ng/ml (normal range: 0-7), which was the upper limit of our laboratory. But he didn't appear jaundice and the initial liver function was Child-Pugh B. The patient underwent abdominal computed tomography (CT), which demonstrated three irregular and low density masses in the liver. These masses located at the top and right lobe of the liver, with a maximum volume of 6 cm × 8 cm × 10 cm (Figure 1A). In addition, portal vein tumor thrombosis was shown on CT (Figure 1B). He was performed liver puncture and confirmed histologically as HCC, C stage (Barcelona Clinic Liver Cancer staging system, 2010) (Figure 2).

**Figure 3:**
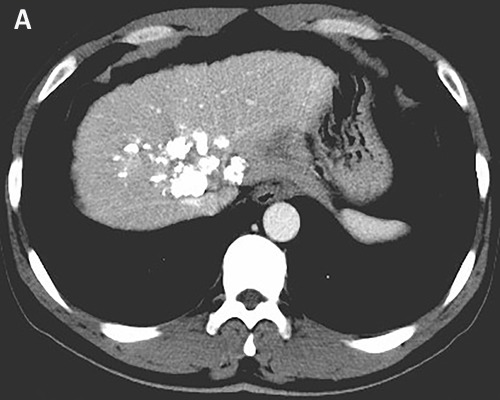

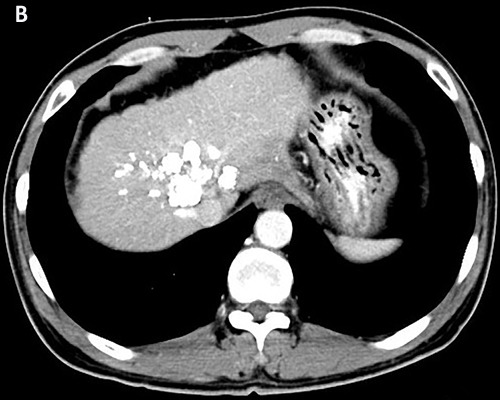
Tumor shrinkage was confirmed CT scan on March 2016 **A**. and on May 2016 **B**. showed that tumor was smaller after using apatinib.

**Figure 4:**
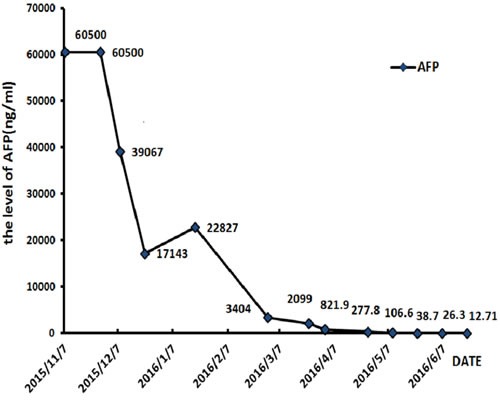
The level of serum AFP keeps falling during treatment

The patient received TACE on day 1, using oxaliplatin 150mg, tegafur 1g, lipiodol 10ml, and took orally apatinib with a dose of 500 mg/d from day 4 to 24. He accepted the treatment for three times once a month from November 2015. The CT scan after therapy exhibited that the lesions were much smaller (Figure 3A). And the serum AFP concentration decreased to 2099 ng/ml (normal range: 0-7). He was evaluated as partial response (PR) by the Response Evaluation Criteria in Solid Tumors (RECIST). Subsequently, the patient received chemotherapy of regimen FOLFOX4, oxaliplatin intravenously at 85 mg/m^2^ on day 1, calcium levofolinate 200 mg/m^2^ on day 1 and 2, 5-fluorouracil 400 mg/m^2^ intravenously and 5-fluorouracil 600 mg/m^2^ intravenously pumped for 22h on day 1 and 2, cycled every two weeks for seven cycles. He took concurrently apatinib with a dose of 500 mg daily from 1 to 10 days per cycle.After the therapy, the CT scan showed the lesions were similar to latest images (Figure 3B), and the serum AFP concentration has been persistently decreasing to 12.7 ng/ml (Figure 4). Finally, the case was confirmed as PR by RECIST.

The patient experienced some toxicities, including hand-foot syndrome (grade 3), diarrhea (grade 2), hypertension (grade 1), decline of leucocyte (grade 1) and platelet (grade 2), and short-lived elevated blood bilirubin and aminotransferase (grade 1). But these adverse effects were controllable and tolerable.

Now, the patient is still taking apatinib as single agent for maintenance therapy with mild toxic effects. And the progression-free survival (PFS) time is more than eight months.

The study was approved by the institutional review board of Radiation Oncology, Shandong Cancer Hospital and Institute. The patient provided written informed consent.

## DISCUSSION

For local and early HCC patients, surgery is the gold-standard treatment, often complemented by interventional therapy or radiofrequency ablation. But, in advanced HCC patients, therapy options are complicated, and the clinical efficacy is always unsatisfactory, due to the relapse, metastasis and patients’ poor performance status. According to the results of the current studies, targeted therapy serves as an important role on the advanced HCC patients, such as sorafenib [5, 6].

Angiogenesis is mediated by vascular endothelial growth factor (VEGF) and act as an important role in the process of tumor growth [7]. When VEGF combines with vascular epidermal growth factor receptor (VEGFR), the downstream signals will be active to stimulate the proliferation of vascular endothelium. VEGFR family proteins are membrane receptor tyrosine kinases, including VEGFR-1, VEGFR-2 and VEGFR-3 [8]. VEGFR-2, which are mainly expressed on endothelial cells, mediates the angiogenic, mitogenic and permeability-enhancing effects of VEGF [9]. It is considered that blockage of VEGFR-2 could be a promising strategy to inhibit tumor-induced angiogenesis.

Apatinib is the latest inhibitor of VEGFR-2 targeting the intracellular ATP-binding site of the receptor, which could inhibit VEGF-stimulated endothelial cell migration and proliferation, decrease tumor microvascular density, and promote apoptosis [10–12]. There are several pre-clinical and clinical trials proving the effect and safety of apatinib. A phase I study was conducted for patients with advanced solid tumors, and turned out that the maximum-tolerated dose was 850 mg once daily [13]. The adverse events of apatinib contained hand-foot syndrome in 46% of patients, proteinuria in almost 50% of patients, and hypertension in about 70% of patients. A phase II study in metastatic gastric cancer patients displayed that apatinib obviously improved PFS comparing with placebo (3.67 months *vs*. 1.40 months) [2]. The toxicities included fatigue, hypertension and hand-foot syndrome, which were well controlled. In patients with advanced or metastatic adenocarcinoma of the stomach or gastroesophageal junction, it was also verified that median overall survival (OS) was significantly improved in the apatinib group compared with the placebo group (6.5 months *vs*. 4.7 months, *P* = 0.0149), the same as PFS (2.6 months *vs*. 1.8 months, *P* < 0.01) [3]. Qin et al. [4] reported that apatinib was efficient for patients with advanced HCC as the first line therapy. In their study, advanced HCC patients were randomized into two groups in which they took apatinib 850 mg or 750 mg daily respectively until progression of the disease. The time to progression (TTP) was 4.21 and 3.32 months in 850 mg and 750 mg groups (*P* > 0.05), respectively. And adverse events were similar in the two groups. They preferred to recommend 750 mg once daily for the next clinical study. Recently, apatinib also shown satisfactory efficacy in non-small cell lung cancer [14], breast cancer [15, 16], malignant fibrous histiocytoma [17], intrahepatic cholangiocarcinoma [11], and extrahepatic bile duct carcinoma [12].

According to the treatment guideline, TACE and chemotherapy are dominating managements for advanced HCC patient. But targeted therapy has been a hot topic in multidisciplinary therapy of HCC. Huang et al. [18] performed a study in intermediate stage HCC patients, and the therapeutic regimen was metronomic S-1 chemotherapy in combination with TACE. The median TTP was 6 months. A phase II study of the combination of TACE and sorafenib in patients with unresectable HCC showed that the median TTP was 5.1 months [19]. Hu et al. compared TACE plus sorafenib with TACE alone in Barcelona Clinic Liver Cancer stage C patients, and the TTP was longer in the combined group (2.6 months *vs*. 1.9 months, *P* = 0.001) [20]. Abou-Alfa et al. [21] analized the difference between sorafenib plus doxorubicin and doxorubicin alone, which showed an apparent improvement in both PFS (6.0 months *vs*. 2.7 months, *P* = 0.006) and OS (13.7 months *vs*. 6.5 months, *P* = 0006). Assenat E et al. [22] compared sorafenib plus gemcitabine/oxaliplatin with sorafenib alone as the first line therapy for patients with advanced HCC. The study declared that DCR and RR were 77% *vs*. 16%, and 70% *vs*. 9%, respectively. A lot of data suggested that targeted therapy combined with TACE or chemotherapy might develop synergetic effects. However, the expensive cost and toxicities of sorafenib limited the utilization. Although apatinib showed notable efficacy in some solid tumors, there is no data about apatinib combined with TACE and chemotherapy in HCC. For this case, based on previous datas of apatinib in HCC, we attempted to administrate him multidisciplinary therapy including apatinib combined with TACE and chemotherapy. In our clinical experience, many gastric cancer patients could not tolerate the toxicity of apatinib alone with the dose of 850 mg daily. In this case, we prescribed him 500 mg daily combined with TACE or chemotherapy. However, he still suffered hand-foot syndrome and diarrhea at the first cycle, and we had to interrupt the medicine for two days. In the follow-up cycles, the adverse events were tolerable gradually and the therapy remained ceaselessly. The patient was evaluated as partial response and the PFS were 8 months until now, which was much longer than that of previous studies [4, 18–21]. This case suggested that the combined therapy of apatinib and TACE followed chemotherapy displayed challenging efficacy. It illuminated that apatinib might cooperate superiorly with TACE and chemotherapy for advanced HCC.

In conclusion, apatinib may provide an additional option for the treatment of liver cancer. Our study indicates that the multiple therapeutics strategies of apatinib combined with other therapies, such as TACE and chemotherapy, should be developed in future clinical trials. Moreover, how to find biomarkers to predict drug efficacy is also one of the challenges with antiangiogenic therapy. Further large-scale prospective studies are required to prove the effect of apatinib in liver cancer.
